# Disruptive Behavior and Factors Associated with Patient Safety Climate: A Cross-Sectional Study of Nurses' and Physicians' Perceptions

**DOI:** 10.1155/2024/5568390

**Published:** 2024-03-12

**Authors:** Pedro Moreno-Leal, César Leal-Costa, José Luis Díaz-Agea, María de los Ángeles Castaño-Molina, María Belén Conesa-Ferrer, Adriana Catarina De Souza-Oliveira

**Affiliations:** ^1^Catholic University of Murcia, Faculty of Nursing, 30107 Guadalupe, Murcia, Spain; ^2^University of Murcia, Faculty of Nursing, 30120 El Palmar, Murcia, Spain

## Abstract

**Background:**

Few studies have analyzed the negative outcomes of disruptive behaviors in the nurse-physician relationship in patient care and their impact on patient safety. These multicausal studies significantly relate to organizational, institutional, and professional attitudinal risk factors.

**Aim:**

Analyze healthcare professionals' perceptions of disruptive behavior and factors associated with patient safety climate in the nurse-physician relationship at the hospital level.

**Methods:**

A multicenter cross-sectional study was conducted with a sample of 370 nurses and physicians assigned to different public hospitals in the Murcia/Spain region, applying the adapted and validated Spanish version of the Nurse-Physician Relationship Scale: Impact of Disruptive Behavior on Patient Care. The analysis used proportions or means (standard deviation (SD)), univariate and multivariate linear regression models, and the chi-square test.

**Results:**

Disruptive behavior was more prevalent in the ICU (81.6%) and the emergency department (67.8%). Professionals indicate that fear of reprisals is the main barrier to the reporting system. Likewise, stress and frustration are more associated with disruptive behavior and influence the safety climate.

**Conclusion:**

Professionals indicate that disruptive behaviors can have a negative impact on clinical outcomes. Age and type of service were identified as the most relevant socio-occupational factors. Stress, frustration, and communication problems are the factors that most influence the safety climate.

## 1. Introduction

The healthcare industry is considered one of the most complex sectors in the world, alongside aviation and nuclear energy. Labor relations within healthcare systems are especially noteworthy, as they contribute to an environment that is more susceptible to risks and failures. Furthermore, the likelihood of failures increases with the complexity of a system [1]. Interprofessional relationships between healthcare professionals are crucial in developing strategies to reduce disruptive behaviors and improve patient safety. Before this investigation, we conducted a systematic review to identify disruptive behaviors in nurse-physician relationships and their impact on patient care [2]. However, we found limited international studies and none conducted within Spain's healthcare domain. This indicates a significant gap in the literature on disruptive behaviors in nurse-physician dynamics. Therefore, further research is necessary to understand healthcare personnel's perceptions of the factors contributing to disruptive behavior and areas that require improvement to prevent such behavior.

There is no consensus on the definition of disruptive behavior and safety climate. Nevertheless, this study aims to contribute to resolving this issue or advancing current knowledge. Concerning disruptive behaviors, we define them as actions that impede interpersonal communication, strain work relationships, and hinder the sharing of crucial information among professionals, thereby directly impacting the quality of the care process [3, 4]. According to the patient safety culture [5], disruptive behaviors can lead to errors in the care process. Our study defines safety climate as how organizational factors influence the safety culture perceived by professionals and institutions [6, 7]. Specifically, patient safety culture is a strategic focal point to encourage healthcare professionals to adopt attitudes and behaviors that encourage patient safety [8]. Moreover, it fosters a nonpunitive environment in which individuals at all levels of an institution or organization (including caregivers, managers, and administrators) pledge to improve patient safety by promoting error reporting as a source of learning rather than blame [9, 10]. Cooper et al. stress the significance of fostering an organizational culture that esteems professionals, caregivers, managers, and administrators who adeptly navigate ethical conflicts impacting the quality of the care process. This culture encompasses effective communication (encompassing behavior management, staff safety status, and attitudes) and procedures (encompassing participation in decision-making, adherence to protocols, and task allocation) [11]. The perception of an unfavorable environment can lead to behaviors associated with horizontal violence, which negatively impacts patient safety [5]. On the other hand, creating a safe environment that promotes an improved safety climate can positively influence professionals' perceptions of workplace safety, leading to more favorable attitudes and behaviors toward patient safety. Research indicates a notable reduction (76%) in adverse event rates associated with such improvements [9, 12].

Many factors that cause disruptive behaviors are closely related to patient safety culture, particularly communication and teamwork. These factors significantly influence compliance with safe work practices [13] and healthcare professionals' perception of a safe environment [14]. Disruptive behaviors may be linked to low job satisfaction due to poor work relationships and co-worker communication [15]. Organizational risk factors at work [16], which include various aspects such as strategies, behavior, and attitudes adopted by healthcare centers to improve the safety environment, can influence professionals' perceptions. The attitudes and approaches of institutional managers and professional burnout can significantly affect emergency nurses' satisfaction and quality of work life.

For a long time, healthcare professionals and institutions did not openly acknowledge disruptive behaviors or measure their impact. In 2001 and 2002, the American Association of Critical Care Nurses (AACN) recognized the need to address the working relationships between nurses and management physicians. They emphasized the importance of establishing a reporting system for disruptive behaviors in healthcare facilities. They stated that such behaviors could not be ignored because they disrupt the workplace and can lead to unpleasant incidents and possible workplace accidents [17, 18]. In 2005, disruptive behaviors were observed to affect patient care and attention [19, 20]. In 2004, the Institute for Safe Medication Practices highlighted potential risks to patient safety due to the approach to medications. They stated that the disruptive behavior of some physicians inhibits nurses from asking questions or providing information about the use of drugs. This behavior is labeled “dangerous silence” and can be interpreted as abusive behavior by some physicians that prevents nurses from answering questions or seeking clarification [21]. In 2008, Rosenstein et al. [22] found that healthcare professionals identified disruptive behaviors as a cause of adverse events that disrupt the chain of patient safety. In 2012, the same author emphasized the connection between disruptive behavior and patient safety. The study revealed that nearly 33% of physicians and nurses believed that these behaviors could lead to adverse events, and, more alarmingly, 12.3% of them were associated with an increased risk of patient mortality [23].

Disruptive behaviors generate vertical workplace violence and are considered a public health problem with global repercussions, affecting the entire healthcare system in its multiple spheres and levels [24]. As part of its healthcare quality accreditation process for healthcare institutions, the Joint Commission has made it mandatory for institutions to implement policies that address disruptive behavior. These policies should be based on human capital prepared to handle the complexity of the healthcare environment. The aim is to prevent and control factors associated with disruptive behavior while ensuring patients' safety and protecting healthcare professionals' occupational health (physical, mental, and emotional) [25]. This study aims to analyze healthcare professionals' perceptions of disruptive behavior and factors associated with patient safety climate in the nurse-physician relationship at the hospital level.

## 2. Materials and Methods

### 2.1. Study Design

A multicenter cross-sectional study was conducted to assess the perceptions of healthcare professionals at the hospital level about disruptive behaviors and factors associated with the patient's safety environment. The research was conducted at the hospital level within the network of public hospitals in the Murcia region. This region encompasses nine referral hospitals, each corresponding to one of the nine Health Area Managements of the Murcian Health Service. Specifically, this study was conducted in five of the nine referral hospitals.

### 2.2. Participants

The sample consisted of nurses (direct care), administrative nurses (indirect care/management), physicians (direct care/management), and administrative physicians (indirect care/management) assigned to different clinical and surgical services.

The study included all physicians and nurses working in public hospitals in the Murcia region, Spain, who met the following criteria: (a) had a contractual relationship (permanent/interim) with any of the hospital services, (b) had worked for more than a year in the service/unit and job position, (c) were Spanish or naturalized citizens and belonged to different work shifts, and (d) agreed to participate in the study. We excluded professionals with temporary contracts or on standby (holidays or temporary leave) because these contracts were subject to short periods in the same department/unit/plant and high staff turnover, which could bias the perception of the safety climate and work relationships.

The professionals were identified based on the hospital's human resources lists.

### 2.3. Procedure

During the study period, from January to July 20, 2022, a researcher was responsible for providing and collecting evaluation instruments. The researcher personally delivered each instrument to participants and explained the importance of their participation in the study. No personal data were included in the instrument to ensure anonymity and confidentiality. Participants were instructed to complete the scales and group them by units/services. The completed scales were collected and sealed in an envelope. In April 2022, a reminder intervention was conducted to increase the response rate.

### 2.4. Measurements

The instrument used was the Spanish version of the original “Survey on the nurse-physician relationship: The impact of disruptive behavior on patient care” [25]. This scale was cross-culturally adapted and validated for use at the hospital care level in Spain. [26]. In this study, the scale obtained a Relevance Index (RI) of 0.89 and a Pertinence Index (PI) of 0.94. The RI and PI values were both below 8, which was considered acceptable for each item and for the scale as a whole. Most of the items in the scale showed a moderate to almost perfect level of concordance between responses (16 items). The Intraclass Correlation Coefficient (ICC) values for these items were equal to or greater than 0.75, indicating excellent reproducibility. Additionally, all items in the scale showed a general agreement index of 100%. The scale is made up of 21 items. In the first part of the scale, the socio-occupational variables are presented: age, sex, service, and position (nurse and clinician or administrative physician). The latter was identified as nurse and clinician, which defines professionals who spend 50% or more of their working day in clinical tasks/direct patient care, and nurse and administrative/managerial physician, which represents professionals who spend 50% or more of their working day in administrative tasks/indirect management. Items 1 to 9 assess the perception of the environment, specifically the safety climate, in the relationship between nurses and physicians, addressing the presence and frequency of disruptive behaviors in different services and specialties. However, items 10 to 17 focuses on assessing the perception of the impact of disruptive behaviors on patient safety, considering various psychosocial aspects, adverse events, and dimensions such as communication and information. Items 18 to 21 focus on assessing the reporting system for disruptive behaviors and barriers that may hinder its effectiveness (Supplementary Table S1). This scale provides a comprehensive measure of the perception of disruptive behaviors in the relationship between physicians and nurses and their impact on patient care.

### 2.5. Data Analysis

Each item on the scale was used as a variable to assess healthcare professionals' experiences of disruptive behavior in the physician-nurse relationship and its impact on patient care. No missing data was present as we discarded incomplete questionnaires. This upheld data integrity for accurate analysis.

Proportions or means (standard deviation (SD)) were used to describe the participants' characteristics and the questionnaire's items. Univariate and multivariate linear regression models were used to analyze the perception of the environment of the physician-nurse relationship and the severity of problems caused by disruptive behavior. The chi-square test was used to compare the proportion of physicians and nurses who had witnessed disruptive behavior. We also used this test to examine the frequency with which physicians and nurses believe disruptive behavior negatively affects the team and patients. *P* values <0.05 were considered significant. All analyses were performed using SPSS software version 22.0 (IBM, Armonk, NY, USA).

### 2.6. Ethical Considerations

Approval was obtained from the Ethics Committee of the Catholic University of Murcia (Code No. CE041825) and from all participating hospitals to conduct the study. Furthermore, confidentiality and data protection are guaranteed by Organic Law 3/2018, of 5 December, on the Protection of Personal Data and the Guarantee of Digital Rights [27].

The Materials and Methods section should contain sufficient detail to repeat all procedures. It may be divided into headings if several methods are described.

## 3. Results

Of the 500 nursing and medical professionals from public hospitals in the Murcia region invited to participate in this study, 370 responded to the scale/instrument (74%). Most of the sample consisted of men (53%) between 20 and 29 years old (42.7%). 41.1% belonged to the emergency department. Regarding position/category, there were few differences in the frequency of participation, except for the low participation of administrative physicians (indirect assistance/management) (18.4%) in the other categories (Table 1).

The average perception of the nurse-physician relationship environment among the 370 participants was 8.05 (SD = 1.59).

Table 2 details the mean values for each variable studied and the results of the univariate and multivariate linear regression analyzes that identify the sociodemographic and occupational determinants of the environment of the nurse-physician relationship. The findings revealed a statistically significant association between the variable age range 30–49 years, both in the univariate (0.487, *p* < 0.05) and multivariate (0.566, *p* < 0.05) models, compared to the reference group (20–29 years). A significant association was also found with the administrative group (indirect care/management) of physicians in the univariate (0.975, *p* < 0.05) and multivariate (0.625, *p* < 0.05) models compared to physicians (direct care). The intensive care unit (ICU) (univariate −0.453, *p* < 0.05; multivariate −0.505, *p* < 0.05) and surgery (univariate −1.090, *p* < 0.001; multivariate −1.078, *p* < 0.001) also showed significant associations compared to the emergency department. Nagelkerke's square *R* indicated that the independent variables used in the multivariate linear regression model explained 11.5% of the variance of the dependent variable.

According to the perception of nurses and physicians (*n* = 370), a higher prevalence of disruptive behaviors was observed in specific areas, the most affected being the intensive care unit (ICU) with 81.6% (*n* = 302), followed by the emergency department with 67.8% (*n* = 251) and general medicine with 58.6% (*n* = 217). Regarding the frequency of such behaviors according to specialty, respondents reported a higher incidence in general surgery with 83.0% (*n* = 307), followed by obstetric/gynecology with 45.9% (*n* = 170), and cardiology with 40.8% (*n* = 151). On the other hand, the specialty with the lowest frequency of disruptive behavior was anesthesia, with 13.2% (*n* = 49) of affirmative responses.

Table 3 shows that physicians (clinical) (87.6%) and administration/management nurses (81.2%) were the most frequent witnesses of disruptive behavior by a physician. When asked, have you ever witnessed disruptive behavior by a nurse at your hospital? A positive response from clinicians was observed (96.6%). Furthermore, compared to nurses, a significant difference was found and nurse clinicians (76.9%, *p* < 0.001) reporting more disruptive behavior from another nurse clinician.

In the multivariate model, the perception of the severity of disruptive behavior problems was primarily influenced by age and the care service.  Table 4 shows that physicians and nurses in the age range between 30 and 49 years and those older than 50 years have a more marked perception compared to other age groups. Furthermore, the surgical service showed a significant influence on this perception in both professional categories, with coefficients of 0.911 (*p* < 0.001) for physicians and 0.674 (*p* < 0.001) for nurses.

When analyzing the impact of the results of disruptive behaviors in the nurse-physician relationship on the patient safety climate, the following factors were identified: stress and frustration (219, 59.2%), loss of concentration (207, 55.9%), reduced teamwork (161, 43.5%), reduced information sharing (214, 57.8%), reduced communication (269, 80.0%), and problems in the nurse-physician relationship (256, 69.2%). When analyzing the differences between physicians and nurses in these factors, it was found that loss of concentration, reduction in transmitted information, and problems in the nurse-physician relationship have a significant implication (*p*  <  0.001) on the patient's safety environment according to nurses compared to physicians (see Table 5).

When asked about the relationship of disruptive behavior with aspects or indicators related to patient safety, the following percentages were identified: adverse events (25.4%), patient safety errors (13.0%), quality of care (20.8%), patient mortality (14.9%), nurse satisfaction (33.2%), physician satisfaction (43.2%), and patient satisfaction (39.2%). When analyzing the differences between physicians and nurses, it was found that physicians have a significantly more negative perception of quality of care (*p* < 0.001) and patient mortality (*p* < 0.001) than nurses.

Most professionals, 83.8% (*n* = 310), indicated that they were aware of a possible adverse event that could have occurred as a result of disruptive behavior. Furthermore, 29.0% (*n* = 90) stated that such events could be severe. Some 47.3% (*n* = 175) indicated that they were aware of the following adverse events that had occurred as a result of disruptive behavior: lack of information (8.6%), delays in care (28.0%), misunderstandings between staff (26.9%), and misinformation provided to relatives (36.6%).

Four questions were asked about the system to prevent and report patient safety incidents. When asked whether incidents could have been prevented, 94.9% (*n* = 166) answered yes. Regarding the conduct procedure, 99.7% (*n* = 369) indicated that a code of conduct or protocol is in place to address disruptive behavior in their hospital. Of these, 27.8% (*n* = 103) stated that a protocol was followed, while 71.9% (*n* = 266) mentioned a code of conduct. Virtually all professionals (99.7%) stated that a nonpunitive recording system was in place for those who witnessed or experienced disruptive behavior. In terms of barriers or obstacles to reporting disruptive behavior, practitioners noted fear of reprisals (82.4%), lack of confidentiality (19.7%), feeling that nothing would change (31.6%), and no response or outcome (10.0%).

## 4. Discussion

Overall, professionals assessed the nurse-physician relationship environment positively, though disruptive behaviors were noted in clinical practice, potentially impacting safety climate and clinical outcomes. Age and service type emerged as key variables affecting perceptions of disruptive behavior impact. Stress, communication barriers, and nurse-physician relationship issues were linked to disruptive behavior. Nurses reported more negatively affected concentration and information transmission. Due to disruptive behavior, physicians perceived lower care quality, safety, and higher mortality rates. Such behaviors also diminished satisfaction among patients, physicians, and nurses. Professionals recognized patient safety incidents associated with disruptive behaviors but did not understand safety incident taxonomy. A cultural perception hindered trust in reporting systems for learning and improvement, indicating a need for cultural change as a priority in improvement strategies.

Although disruptive behaviors are not uncommon [28, 29] and should be of concern for healthcare institutions to improve patient safety and foster a working environment conducive to positive outcomes [7, 30], few studies have been published on this problem in the healthcare setting. This is the first study in Spain, to our knowledge, that explicitly addresses disruptive behaviors in the healthcare setting. The first published studies correspond to Rosenstein et al., the authors of the instrument used in our research. In 2002, they analyzed 1,200 questionnaires on the United States West Coast [25]; in 2005, there were 244 participants [31].

In recent years, studies like ours have obtained a lower response rate than ours (74%). For example, a study in Singapore had a response rate of almost 40% (39.9%), and most of the respondents were physicians (64.2%) [32]. This contrasts with our results. In the context of Iranian healthcare care, we found two relevant studies. One of them, carried out in health centers affiliated with the University of Isfahan, involved 248 professionals, most of them nurses [33]. The other study was carried out in four emergency departments, with 45 physicians and 110 nurses responding [34]. Considering cultural and social differences, the professionals participating in our study may have a greater postpandemic awareness, leading them to participate in studies to improve the psychosocial aspects associated with the care process.

According to our 10-point maximum rating scale, our professionals reveal a moderate-high degree (with an average of 8.05 points). Being between 30 and 49 years old and working in the surgery and ICU departments are the sociodemographic and occupational factors most influencing this perception. Regarding age, these results were expected, as it is likely that, with increasing age, professionals acquire more experience and a more critical view of their working environment, identifying aspects that may go unnoticed by their younger colleagues.

Regarding the type of service, several studies have found that emergency and operating room areas are the most significant in the manifestation of disruptive behavior [22, 23, 25, 33, 35]. These two environments are high-stress environments characterized by high communication flow and remarkable concentration. Surprisingly, our findings, in agreement with those of Rosenstein and O'Daniel [35], indicate that the emergency department is not significant in overall perception; on the contrary, the intensive care unit (ICU) and the operating theatre are. The Nagelkerke *R* square coefficient of determination value of 11.5% highlights the importance of interpreting this result with caution and assessing the linear relationship with other socio-occupational variables in future research.

In terms of the type of department and specialty, there are different perceptions. When asked about the prevalence of disruptive behaviors by department type, the emergency department and the ICU are the most relevant in our study. However, in terms of specialty, they are more frequent in general surgery, which coincides with the study by Saghaei et al. [33]. This reflects that these services have a context characterized by high demand and a high level of technology where life and death are separated by an instant or an error in care. It is understood that these characteristics can contribute to the perception of disruptive behaviors in these environments.

Our results reveal a significant discrepancy with the existing literature on the observation of disruptive behaviors. Previous research has indicated that clinicians and nurse clinicians frequently witness such behaviors in their work environments, primarily by clinicians. However, in our study, clinicians reported seeing disruptive behavior from other physicians and nurses with greater frequency than that reported by nurses, in line with the results of Lim et al. This finding is remarkable and contradicts the prevailing conception, suggesting that direct care nurses, who work in contexts characterized by hierarchies, manifestations of authority, and negotiation of responsibilities, especially in emergency and operating rooms, are more susceptible to abusive behaviors from physicians.

Both older physicians and nurses show a higher perception of the severity associated with disruptive behaviors, with significant negative implications in the context of the surgical service. This phenomenon suggests that these factors are relevant in the professional assessment of the seriousness of disruptive behaviors. This finding indicates that more experienced practitioners may be more willing to express their views on disruptive behaviors' possible complications and effects.

According to the perspective of professionals and according to the existing literature, the main factors linked to disruptive behaviors that impact the safety climate include stress and frustration [32–39], poor communication [32], and problems in the nurse-physician relationship [33]. However, nurses report a more negative perception of lost concentration and reduced information transmission than their medical colleagues. This insight underscores the importance of communication and information for safer care [40, 41]. Professionals recognize the relevance of all aspects of communication for continuity of care and to promote a positive working relationship between nurses and physicians [42]. Both healthcare bodies and international organizations recognize that deficiencies in patient information transmission can cause substantial safety problems [42, 43]. Effective communication is a global goal to improve patient safety [44], as reflected in Strategic Objective 6: Information, research, and risk management of the World Patient Safety Action Plan 2021–2030 [45]. According to Astier-Peña et al. [46], this goal aims to ensure a better flow of information and knowledge to promote risk management and ensure more respectful care at all levels of care.

Regarding the undesirable clinical outcomes associated with disruptive behaviors, professionals point out that these directly impact the satisfaction levels of patients and professionals themselves, according to previous research [32–34, 47]. There is also evidence of their relationship with adverse events in clinical practice. Given the consequences and impact of disruptive behaviors, these results were predictable. The degree of satisfaction is not always determined solely by the structure or level of knowledge; it can be related to a culture of attitudes and behaviors that have a negative impact on working relationships [48, 49], compromising the safety climate, weakening teamwork, and affecting job satisfaction. Furthermore, our findings highlight that physicians are the ones who most strongly perceive the relationship between disruptive behaviors and poor quality of care and patient mortality, in agreement with another research [32, 34]. However, these cause-effect results must be assessed with caution, as other factors that have not been studied or may be intrinsically or hidden in negative behaviors and attitudes can be involved, which can be detrimental to the care process.

Although unwanted events due to disruptive behavior were not unexpected, as identified in other studies [20, 22, 25, 32, 34, 41, 45], we were surprised by the high percentage observed in the investigated context. Professionals reported adverse events such as “misinformation to relatives,” “delay in care,” and “misunderstandings between staff.” We recognize that disruptive behaviors affect the safety climate and can have severe consequences on the job, compromising the nurse-physician relationship and creating obstacles to improving the quality of care. However, when examined from the perspective of the taxonomy of safety incidents proposed by the Heinrich Pyramid, we observe that, rather than events, they constitute patient safety incidents with the potential to cause patient harm [50]. These incidents are classified as near misses, indicating the possibility of having caused harm to the patient [50], and physicians indicated that these risk circumstances for patient safety could have been avoided. Furthermore, they noted clear guidelines in their centers on addressing disruptive behaviors, through protocols or codes of conduct. We believe that this aspect is relevant and should be integrated into the healthcare management strategies of each center and institution.

In examining the question related to the reporting system for disruptive behaviors witnessed or experienced, almost all practitioners indicated that it was a nonpunitive system. However, a significant proportion of them expressed that fear of reprisals was a major concern, acting as a substantial barrier to reporting such behavior. Furthermore, they reported a perceived lack of feedback or positive response as a consequence of the report. They noted that there was no change in practice, findings that are consistent with previous research [31, 33, 34, 47]. Against this backdrop, several questions arise. Is there truly a nonpunitive system, or does fear persist among professionals to speak openly and honestly about the reporting system? Do professionals understand the inherent meaning and function of a reporting system? Have health institutions succeeded in effectively implementing a reporting system? These questions raise fundamental questions about the culture of patient safety. Despite more than two decades since the publication of the report To Err Is Human [51], it remains imperative to address these issues to drive continuous improvement in quality and safety in healthcare. The foundation of all healthcare systems is an awakening towards improving patient safety, evidenced by joint efforts and focused attention on this crucial aspect. Despite two decades since the National Quality Forum's recommendation to implement safety culture as the first of its “30 safe practices,” there is still a way to go towards fully realizing this goal [52]. From our perspective, the “tip of the iceberg” represents only a visible fraction of a broader set of factors influencing or determining safe practice. We recognize that visible and invisible aspects intrinsically relate to patient safety culture. This culture, characterized by its nonpunitive nature and its focus on learning from mistakes, is a fundamental element in promoting safety and improving the quality of care [8].

This study is not without limitations. First, the sample used. Our study focused on five hospitals of the 9 Health Departments of Murcia Healthcare, Spain. This selection can restrict the interpretation of the results, as it is described as a global perception of professionals only in hospitals in a specific region of Spain. Although it was not our main objective, it is important to note that including other professional categories could enrich the understanding of the general importance of disruptive behaviors. This aspect should be addressed in future studies. It is essential to remember that the subjects in our study represent only a sample of the total population, which also implies certain limitations regarding the generalizability of the findings.

The second aspect refers to the “Nurse Physician Relationship Survey: Impact of Disruptive Behavior on Patient Care.” Although the results of the previous study of adaptation and validation [24] in Spanish were satisfactory, certain important aspects must be considered. Not many questionnaires or scales have been found that specifically address disruptive behavior in the hospital setting. Although this scale covers all the issues relevant to our research objectives, few studies are available to compare the results obtained. In the Spanish context, none have been identified to date. More research is needed to assess the perception of disruptive behaviors in the hospital setting and their impact on patient safety using this national and international instrument to establish meaningful comparisons between different countries.

Finally, another study limitation is the lack of consideration for potential confounding variables. While efforts were made to control for known factors, variables beyond the scope of this research could influence outcomes. Future studies should address these variables to provide a more comprehensive understanding of the phenomena under investigation.

## 5. Conclusions

Professionals have assessed that the nurse-physician relationship environment is relatively good overall. However, disruptive behaviors have been observed in clinical practice, which can have a negative impact on the safety climate and clinical outcomes.

Age and type of service were the most relevant socio-occupational variables for the perception of the impact of disruptive behavior in the nurse-physician relationship. The factors most associated with disruptive behavior and influencing the safety environment included stress and frustration, reduced communication, and problems in the nurse-physician relationship. Nurses expressed significantly more negative perceptions of losing concentration and reducing information transmission.

Regarding the impact of disruptive behaviors on the nurse-physician relationship and clinical outcomes, physicians have a more unfavorable perception of quality of care, patient safety, and even mortality rate. In addition, disruptive behaviors negatively influence patient, physician, and nurse satisfaction.

We have observed that professionals do not yet understand the taxonomy of patient safety incidents, but they have a relatively high perception of incidents associated with disruptive behaviors. In addition, a cultural perception persists that generates fear and “low credibility” with respect to the reporting system as a tool for learning and improvement. Changing culture is not an easy challenge, but it significantly impacts other countries and remains a priority in improvement strategies.

## Figures and Tables

**Table 1:** Characteristics of the participants.

Variables	*n* (%)
*Sex*	
Woman	174 (47.0)
Men	196 (53.0)

*Age*	
20–29 years	158 (42.7)
30–49 years	121 (32.7)
>50 years	91 (24.6)

*Job position*	
Physician (clinical)	97 (26.2)
Physician (administrative)	68 (18.4)
Nurse (clinical)	101 (27.3)
Nurse (administrative)	104 (28.1)

*Unit*	
Emergency department	152 (41.1)
Intensive care unit (ICU)	137 (37.0)
Surgery	81 (21.9)

**Table 2:** Univariate and multivariate analysis of the perception of the environment of the nurse-physician relationship.

Variables	Mean (SD)	Univariate B coefficient (SE)	Multivariate B coefficient (SE)
*Sex*			
Woman	7.91 (1.56)	Reference	
Men	8.18 (1.61)	0.265 (0.166)	

*Age*			
20–29 years	7.84 (2.03)	Reference	
30–49 years	8.32 (1.29)	0.487 (0.191)^*∗*^	0.566 (0.171)^*∗*^
>50 years	8.08 (1.00)	0.241 (0.208)	

*Job position*			
Physician (clinical)	7.70 (1.67)	Reference	
Physician (administrative)	8.68 (1.55)	0.975 (0.248)^*∗*^	0.625 (0.206)^*∗*^
Nurse (clinical)	8.03 (1.49)	0.329 (0.223)	
Nurse (administrative)	8.00 (1.53)	0.299 (0.221)	

*Unit*			
Emergency department	8.46 (1.71)	Reference	
Intensive care unit (ICU)	8.01 (1.08)	−0.453 (0.182)^*∗*^	−0.505 (0.183)^*∗*^
Surgery	7.37 (1.82)	−1.090 (0.212)^*∗∗*^	−1.078 (0.212)^*∗∗*^

Nagelkerke *R* Square: 0.115. ^*∗*^*p* < 0.05; ^*∗∗*^*p* < 0.001.

**Table 3:** Frequency of witnessing disruptive behavior.

Job position	Have you ever witnessed disruptive behavior from a physician in your hospital?	
	Yes	No
Physician (clinical)	85 (87.6)	12 (12.4)
Physician (administrative)	53 (77.9)	15 (22.1)
*p* value		0.098
Nurse (administrative)	82 (81.2)	19 (18.8)
Nurse (clinical)	77 (74.0)	27 (26.0)
*p* value		0.220

	Have you ever witnessed disruptive behavior from a nurse in your hospital?	

Physician (clinical)	94 (96.9)	3 (3.1)
Physician (administrative)	63 (92.6)	5 (7.4)
*p* value		0.210
Nurse (administrative)	55 (54.5)	46 (45.5)
Nurse (clinical)	80 (76.9)	24 (23.1)
*p* value		<0.001

**Table 4:** Perception of the severity of problems caused by disruptive behavior.

Variables	Physicians	Model 1	Model 2	Nurses	Model 3	Model 4
*Sex*						
M	2.83 (1.77)	Reference		2.37 (1.27)	Reference	
W	2.47 (1.44)	−0.353 (0.168)^*∗*^	−0.377 (0.156)^*∗*^	2.13 (1.10)	−0.246 (0.124)^*∗*^	

*Age (years)*						
20–29	3.02 (1.90)	Reference		2.28 (1.24)	Reference	
30–49	1.97 (1.26)	−1.052 (0.187)^*∗∗*^	−1.073 (0.166)^*∗∗*^	1.93 (1.17)	−0.359 (0.141)^*∗*^	−0.343 (0.138)^*∗*^
>50	2.88 (1.14)	−0.140 (0.204)		2.59 (1.02)	0.309 (0.154)^*∗*^	0.433 (0.152)^*∗*^

*Job position*						
Phys. (C)	3.22 (1.99)	Reference		2.37 (1.35)	Reference	
Phys. (A)	2.75 (1.77)	−0.466 (0.249)		2.32 (1.20)	−0.048 (0.187)	
Nurse (A)	2.35 (1.15)	−0.870 (0.224)^*∗∗*^		2.38 (1.07)	0.005 (0.168)	
Nurse (C)	2.32 (1.33)	−0.899 (0.223)^*∗∗*^		1.94 (1.09)	−0.429 (0.167)^*∗*^	

*Servicio*						
ED	2.28 (0.97)	Reference		2.13 (1.01)	Reference	
ICU	2.63 (1.64)	0.345 (0.185)		2.11 (1.21)	−0.016 (0.138)	
Surgery	3.33 (2.22)	1.050 (0.216)^*∗∗*^	0.911 (0.188)^*∗∗*^	2.69 (1.36)	0.566 (0.161)^*∗∗*^	0.674 (0.146)^*∗∗*^

Data are presented as the coefficient b (standard error). Model 1: Univariate model of disruptive behavior of physicians; Model 2: Multivariate model of disruptive behavior of physicians; Model 3: Univariate model of disruptive behavior of nurses; Model 4: Multivariate model of disruptive behavior of nurses. Nagelkerke *R* square: Model 2 = 0.157; Model 4 = 0.098. ^*∗*^*p* < 0.05; ^*∗∗*^*p* < 0.001.

**Table 5:** How often do you think disruptive behavior results in the following? Difference between physicians and nurses.

Impacts	Physicians	Nurses	*p* value
*Stress and frustration*			
No	77 (52.3)	72 (47.7)	0.013
Yes	86 (39.3)	133 (60.7)	

*Loss of concentration*			
No	89 (54.6)	74 (45.4)	<0.001
Yes	76 (36.7)	131 (63.3)	

*Reduced teamwork*			
No	82 (39.2)	127 (60.8)	0.018
Yes	83 (51.6)	78 (48.4)	

*Reduced information transmission*			
No	107 (69.0)	48 (31)	<0.001
Yes	58 (27.1)	156 (72.9)	

*Reduced communication*			
No	36 (48.6)	38 (51.4)	0.433
Yes	129 (43.6)	167 (56.4)	

*Nurse-physician relationship problems*			
No	66 (57.9)	48 (42.1)	<0.001
Yes	99 (38.7)	157 (61.3)	

## Data Availability

The data used to support the study are available from the corresponding author upon request.

## Supplementary Materials

Supplementary Materials Table S1: original and cross-culturally adapted and validated in a Spanish context of the instrument “Survey on the nurse-physician relationship: The impact of disruptive behavior on patient care.”
