# Supplementary material for: Disruptive Behavior and Factors Associated with Patient Safety Climate: A Cross-Sectional Study of Nurses' and Physicians' Perceptions
Source: J Nurs Manag. 2024 Mar 12;2024:5568390. doi: 10.1155/2024/5568390 (PMC11918647; doi:10.1155/2024/5568390)
Supplement: Supplementary Materials — Table S1: original and cross-culturally adapted and validated in a Spanish context of the instrument “Survey on the nurse-physician relationship: The impact of disruptive behavior on patient care.” [file 5568390.f1.docx]

**Supplementary Material**

Table S1. Original and cross-culturally adapted and validated in a Spanish context of the instrument "Survey on the nurse-physician relationship: The impact of disruptive behavior on patient care".

| **Original version** | **Adapted and validated version** |
| --- | --- |
| Title: Nurse-Physician Relationship Survey: Impact of Disruptive Behavior on Patient Care | Título: Escala sobre la relación Enfermero/a - médico: impacto del comportamiento disruptivo en la atención al paciente |
| “Disruptive behavior” is defined as any inappropriate behavior, confrontation or conflict ranging from verbal abuse to physical or sexual harassment. One potential consequence of disruptive behavior is its effect on collaboration and communication between physicians and nurses that may result in an adverse effect on patient care. The current survey is designed to assess the potential impact of disruptive behavior on adverse events, medical errors, patient safety, quality and other outcomes of care. | El “Comportamiento disruptivo” se define como cualquier comportamiento inapropiado, enfrentamiento o conflicto que puede ir desde el abuso verbal hasta el físico o acoso sexual. Una de las potenciales consecuencias del comportamiento disruptivo son sus efectos en la colaboración y comunicación entre los médicos y enfermeras que pueden conllevar resultados negativos en la atención al paciente. El presente cuestionario ha sido diseñado para identificar el potencial impacto del comportamiento disruptivo en los eventos adversos, errores médicos, seguridad de paciente, calidad y otros aspectos relacionados con la atención. |
| Please choose only one answer for each question unless otherwise stated.  If you are completing the survey electronically, you can double click on any box and a prompt will appear. Under default value, click on “checked” and an X will appear in the box. | Por favor, escoja solo una de las respuestas por cada pregunta, salvo que se indique lo contrario. En caso de contestar el cuestionario de forma electrónica, puede hacer doble clic en cualquier casilla y aparecerá un aviso de confirmación.  Debajo de cada valor, haga clic sobre la respuesta y una X aparecerá en la casilla |
| (Survey Demographics: Please choose one in each category) | (Variables sociodemográficas: Por favor, seleccione una por cada categoría) |
| Clinical is defined as 50% or more of time spent with clinical duties | Clínico: se define a los profesionales que pasan el 50% o más de su jornada en tareas clínicas |
| Executive is defined as 50% or more of time spent with administrative duties | Administrativo: se define a los profesionales que pasan el 50% o más de su jornada en tareas administrativas |
| Title | Cargo |
| Physician (Clinical) | Médico (Clínico) |
| Physician (Executive) | Médico (Administrativo/gestión) |
| RN (Executive) | Enfermero/a (Administrativo/gestión) |
| RN (Clinical) | Enfermero/a (Clínico) |
| Administration | Administración |
| Other ____________ | Otros |
| Service | Servicio |
| Medical Service | Centro de salud |
| Emergency Department | Urgencias |
| Intensive Care | Cuidados intensivos |
| Surgical Services | Cirugía |
| Other __________ | Otros |
| Demographics | Sociodemográfica |
| 19 Years or Younger | 19 años o menos |
| 20-29 Years | 20-29 años |
| 30-39 Years | 30-39 años |
| 40-49 Years | 40-49 años |
| 50-59 Years | 50-59 años |
| 60 Years or Older | 60 años o más |
| Male | Hombre |
| Female | Mujer |
| 1) On a scale of 1 – 10 with 10 being the most positive, how would you describe the overall atmosphere of nurse-physician relationships at your hospital? | 1) En una escala del 1 al 10, siendo 10 la más positiva, ¿cómo describiría el ambiente de la relación Enfermera – Médico en su hospital? |
| Very negative | Muy negativa |
| Barely Positive | Poco Positiva |
| Somewhat Positive | Algo positiva |
| Mostly Positive | Bastante positiva |
| Very Positive | Muy positiva |
| 2) Have you ever witnessed disruptive behavior from a physician at your hospital? | 2) ¿Alguna vez ha presenciado comportamiento disruptivo por parte de un/a médico de su hospital? |
| 3) Have you ever witnessed disruptive behavior from a nurse at your hospital? | 3) ¿Alguna vez ha presenciado comportamiento disruptivo por parte de un/a enfermero/a de su hospital? |
| 4). Are there any particular settings where disruptive behavior is most prevalent? (Check all that apply) | 4) ¿Existe alguna unidad en la que el comportamiento disruptivo es más prevalente? (marque todas las áreas) |
| ICU | UCI |
| OR | Quirófanos |
| ED | Urgencia |
| OB | Obstetricia |
| Med unit | Med. general |
| surg. Unit | Cirugía |
| SNF | Enfermería |
| Other | Otros |
| 5) Are there any particular specialties where disruptive events occur most  often? (Check all that apply) | 5) ¿Existe alguna especialidad donde se produzcan comportamientos disruptivos de forma frecuente? (Seleccione todas las especialidades) |
| General Surgery | Cirugía General |
| Cardiac Surgery | Cirugía Cardiaca |
| Cardiology | Cardiología |
| Orthopedics | Ortopédica |
| Neurosurgery | Neurocirugía |
| Anesthesia | Anestesia |
| OB/Gyn | Obstetricia / Ginecología |
| Other | Otros |
| 6) What percentage of physicians would you say exhibit disruptive behavior at your hospital? | 6) ¿Qué porcentaje de médicos diría que muestran comportamiento disruptivo en su hospital? |
| None | Cero |
| More than 10% | Más del 10% |
| 7) What percentage of nurses would you say exhibit disruptive behavior at your hospital? | 7) ¿Qué porcentaje de enfermeros/as diría que muestran comportamiento disruptivo en su hospital? |
| None | Cero |
| More than 10% | Más del 10% |
| 8) How often does physician disruptive behavior occur at your hospital? | 8) ¿Con qué frecuencia los médicos presentan comportamientos disruptivos en su hospital? |
| Daily | Diariamente |
| Weekly | Semanalmente |
| 1-2 Time/Month | 1-2 veces al mes |
| 1-5 Times/Year | 1-5 veces al año |
| Never | Nunca |
| 9) How often does nurse disruptive behavior occur at your hospital? | 9) ¿Con qué frecuencia los enfermeros/as presentan comportamientos disruptivos en su hospital? |
| Daily | Diariamente |
| Weekly | Semanalmente |
| 1-2 Time/Month | 1-2 veces al mes |
| 1-5 Times/Year | 1-5 veces al año |
| Never | Nunca |
| 10) On a scale of 1 – 10 with 10 being the most serious, how serious of an issue is physician disruptive behavior at your hospital? | 10) En una escala del 1 – 10 siendo 10 los casos más graves, ¿Cómo de graves son los problemas causados por el comportamiento disruptivo de los médicos en su hospital? |
| Not Serious | Nada graves |
| Minimally Serious | poco graves |
| Somewhat Serious | Algo graves |
| Mostly Serious | Bastante graves |
| Very Serious | Muy graves |
| 11) How serious of an issue is nurse disruptive behavior in your hospital? | 11) ¿Cómo de graves son los problemas causados por el comportamiento disruptivo de enfermeros/as en su hospital? |
| Not Serious | Nada graves |
| Minimally Serious | Mínimamente graves |
| Somewhat Serious | Algo graves |
| Mostly Serious | Bastante graves |
| Very Serious | Muy graves |
| 12) From your perspective, do you think that disruptive behavior can potentially have a negative effect on patient outcomes? | 12) Desde su punto de vista ¿Cree que el comportamiento disruptivo puede tener efectos potencialmente negativos en la atención al paciente? |
| 13) How often do you think disruptive behavior results in the following?  (never-rarely-sometimes-frequent-constant) | 13) ¿Con qué frecuencia cree que comportamiento disruptivo influye en los siguientes aspectos?  (nunca - en raras veces - algunas veces-frecuentemente - constantemente) |
| Stress | Estrés |
| Frustration | Frustración |
| Loss of concentration | Pérdida de concentración |
| Reduced team collaboration | Trabajo en equipo reducido |
| Reduced information transfer | Información transmitida reducida |
| Reduced communication | Comunicación reducida |
| Impaired RN-MD relations | Problemas en la relación Enfermera – Médico |
| 14) How often do you think there is a link between disruptive behavior and the following?  (never-rarely-sometimes-frequent-constant) | 14) ¿Con que frecuencia considera que existe una relación entre el comportamiento disruptivo y los siguientes aspectos?  (nunca - en raras veces - algunas veces-frecuentemente - constantemente) |
| Adverse Events | Eventos Adversos |
| Errors | Errores |
| Patient safety | Seguridad del paciente |
| Quality of care | Calidad de la atención |
| Patient mortality | Mortalidad del paciente |
| Nurse satisfaction | Satisfacción de los/as enfermeros/as |
| Physician satisfaction | Satisfacción de los médicos |
| Patient satisfaction | Satisfacción del paciente |
| 15) Are you aware of any potential adverse events that could have occurred from disruptive behavior? | 15) ¿Tiene conocimiento de cualquier evento adverso potencial que pudiera haber ocurrido debido un comportamiento disruptivo? |
| 16) If yes, how serious an impact do you think this could have had on patient outcomes? | 16) Si la respuesta es sí, ¿Cómo de grave hubiera sido el impacto en los pacientes? |
| Not Serious | Nada grave |
| Somewhat Serious | Algo grave |
| Serious | Grave |
| Very Serious | Muy grave |
| Extremely Serious | Extremadamente grave |
| 17) Are you aware of any specific adverse events that did occur as a result of disruptive behavior? | 17. ¿Conoce los eventos adversos que han ocurrido como resultado del comportamiento disruptivo? |
| 17.1) If yes, please describe: | 17.1) Si la respuesta es sí, por favor descríbalos: |
| 17.2) Could this have been prevented? | 17.2) ¿Este se podría haber prevenido? |
| 17.3) If yes, please describe: | 17.3) Si la respuesta es sí, por favor descríbalo: |
| 18) Is there a code of conduct or policy for the handling of disruptive/ abusive behavior at your hospital? | 18) ¿Existe algún protocolo para actuar frente al comportamiento disruptivo en su hospital? |
| 18.1) If yes, please explain: | 18.1) Si la respuesta es sí, por favor explíquelo: |
| 18.2) Is the plan effective? | 18.2) ¿El protocolo es efectivo? |
| 18.3) Please explain: | 18.3) Por favor explíquelo: |
| 19) If you know of physicians who have been counseled about his or her behavior, on a scale of 1-10 with 10 being completely satisfied, rate the success of this process. | 19) Si conoce médicos que han sido asesorados debido a su comportamiento, en una escala de 1-10 siendo 10 completamente satisfecho, califique el éxito del proceso |
| Not Satisfied | Nada satisfecho |
| Minimally Satisfied | Poco satisfecho |
| Somewhat Satisfied | Algo satisfecho |
| Mostly Satisfied | Muy Satisfecho |
| Completely Satisfied | Completamente Satisfecho |
| a) If you know of nurses who have been counseled about his or her behavior, on a scale of 1-10 with 10 being completely satisfied, rate the success of this process. | a) Si conoce enfermeros/as que han sido asesorados/as sobre su comportamiento, en una escala de 1-10 siendo 10 completamente satisfecho, califique el éxito del proceso. |
| Not Satisfied | Nada satisfecho |
| Minimally Satisfied | Poco satisfecho |
| Somewhat Satisfied | Algo satisfecho |
| Mostly Satisfied | Muy Satisfecho |
| Completely Satisfied | Completamente Satisfecho |
| 20) Is there a non-punitive reporting environment for those who witness/experience disruptive behavior? | 20) ¿Existe un servicio de registro para aquellos que presencian / experimentan comportamiento disruptivo? |
| 21) Are there any barriers or resistance to the reporting of disruptive behavior? (Check all that apply): | 21) ¿Existe alguna barrera u obstáculo para notificar un comportamiento disruptivo? (Seleccione todos los aspectos): |
| Fear of Retaliation | Miedo a las represalias |
| Lack of Confidentiality | Falta de confidencialidad |
| The feeling that “Nothing ever changes” | La sensación de que “nada cambiara nunca” |
| No feedback of results | No existe respuesta o resultados |
| Other | Otros |
| Please explain: | Por favor explíquelos: |
| Additional Comments (use additional space as necessary) | Comentarios adicionales (utilice el espacio adicional en caso de ser necesario) |
| Your Title (optional) | Su cargo en el trabajo (opcional) |
